# Butaphosphan and Cyanocobalamin Supplementation in Semen Extender on Chilled Boar Sperm Quality and Life Span

**DOI:** 10.3389/fvets.2020.592162

**Published:** 2020-12-01

**Authors:** J. Suwimonteerabutr, S. Chumsri, P. Tummaruk, Morakot Nuntapaitoon

**Affiliations:** ^1^Department of Obstetrics, Gynaecology and Reproduction, Faculty of Veterinary Science, Chulalongkorn University, Bangkok, Thailand; ^2^Swine Reproduction Research Unit, Chulalongkorn University, Bangkok, Thailand

**Keywords:** butaphosphan, chilled boar semen, cyanocobalamin, life span, sperm quality

## Abstract

The objective of the present study was to determine the effect of butaphosphan and cyanocobalamin supplementation in semen extender on chilled boar sperm quality and life span. A total of 35 ejaculates of boar semen were included. The semen was diluted with Beltsville thawing solution extender supplemented with different concentrations of butaphosphan and cyanocobalamin [0 (control), 0.1, 0.2, 0.3, 0.4, and 0.5%] in the diluted semen. The semen samples were evaluated using a computer-assisted sperm analysis system to determine sperm motility and sperm kinetic parameters (i.e., the curvilinear velocity, VCL; straight line velocity, VSL; average path velocity, VAP; linearity, LIN; straightness, STR; amplitude of lateral head, ALH; wobble, WOB; and beat cross frequency, BCF). Additionally, sperm viability, acrosome integrity, mitochondrial activity, and plasma membrane integrity were evaluated after 4 (day 0), 72 (day 3), 120 (day 5), and 168 (day 7) h of storage using SYBR-14–ethidium homodimer-1 (EthD-1), EthD-1, JC-1, and the short hypo-osmotic swelling test, respectively. The analyses were carried out by using the general linear mixed model (MIXED) procedure of SAS. The statistical models for each data set included group, day after storage, and interaction between group and day after storage. The boar was included as a random effect. On day 0 after storage, progressive motility, VCL, VSL, VAP, and plasma membrane integrity of boar sperm in 0.3% of butaphosphan and cyanocobalamin supplementation were greater than those in the 0.4 and 0.5% groups (*P* < 0.05). On day 3 after storage, total motility and progressive motility, VCL, VSL, VAP, LIN, WOB, BCF, and plasma membrane integrity in 0.3% of butaphosphan and cyanocobalamin supplementation were significantly greater than those in the control group (*P* < 0.05). The total motility and progressive motility, VAP, and WOB in 0.3% of butaphosphan and cyanocobalamin supplementation were greater than those in the control group on day 5 after storage (*P* < 0.05). No effects of butaphosphan and cyanocobalamin supplementation on acrosome integrity and mitochondria activity were found on days 3, 5, and 7 after storage. However, the motility and progressive motility and the values for all sperm kinetic parameters except ALH in 0.3% of butaphosphan and cyanocobalamin supplementation were greater than those in the control group on day 7 after storage (*P* < 0.05). In conclusion, 0.3% of butaphosphan and cyanocobalamin supplementation in semen extender improved sperm motility, sperm activity, morphology, and life span in chilled boar sperm.

## Introduction

Artificial insemination (AI) mostly contributes to improved genetics in the modern commercial swine farm. AI reduces the risk of reproductive disease transmission from direct contact between the boar and dam. Moreover, collected semen from pathogen-free boar in the AI unit mainly reduced the risk for introduction or transmission of boar pathogens in the sow herd (1). Over the last decades, chilled boar semen has considerably increased worldwide swine production due to inexpensive and high ratios of the number of boars per sow, reduced stockmanship, and the high impact of reproductive performance in farms, compared to natural mating (2). The intrinsic quality in chilled boar semen influences swine fertility (3). Moreover, fertility rate and litter size positively correlate with sperm kinetic parameters assessed by the computer-assisted sperm analysis (CASA) system and sperm morphology (3). Sperm activity must use energy from the mitochondria in the form of adenosine triphosphate (ATP) from the mitochondria in the midpiece of the sperm for movement and fertilization in the sow's reproductive tract.

Phosphorus is a crucial part of the energy [i.e., ATP and adenosine monophosphate (AMP), creatinine, nucleotide, and glucose production (4)] and important for the metabolism of sperm energy (5). Cyanocobalamin is a cofactor of the methylmalonyl-CoA mutase which is an enzyme used in the conversion of propionate to succinyl-CoA in the Krebs cycle and related to gluconeogenesis (6, 7). Moreover, cyanocobalamin is an antioxidant which reduces free radicals in the body including in sperm production (8, 9). In general, an intramuscular injection of butaphosphan and cyanocobalamin combination reduces the adverse effects from using dexamethasone in dogs, reduces ketosis in dairy cows, improves the energetic status in postpartum ewes, and increases sperm motility in horses (10–14). Therefore, the combination of butaphosphan and cyanocobalamin may be associated with increased sperm energy supply, enhanced sperm membrane stability, and reduced oxidative agents in boar semen. It is currently unknown whether butaphosphan and cyanocobalamin supplementation in chilled boar semen has an influence on sperm quality and life span. Therefore, the objective of the present study was to determine the effect of butaphosphan and cyanocobalamin supplementation on sperm quality and life span in chilled boar semen.

## Materials and Methods

The present study was approved by the Chulalongkorn University Animal Care and Use Committee (animal use protocol number 1831110). The protocols followed the guidelines documented in the ethical principles and guidelines for the use of animals for scientific purposes published by the National Research Council of Thailand.

### Animal

The present study was performed in an evaporative cooling system of a commercial swine herd located in the western part of Thailand. A total of 35 ejaculates of semen from 16 Duroc, 9 Landrace, and 10 Yorkshire boars aged between 1 and 3 years were included in the experiment. The experiment was conducted between May 2019 and February 2020. Boars were kept in individual pens (2.5 × 2.5 m) on a slatted floor. Boars had access to water *ad libitum* and were fed a commercial lactation diet twice a day. Composition and nutrient followed the nutrient recommendations from the NRC (15).

### Semen Collection

The semen was collected by using the gloved-hand method. The boars were allowed at least 7 days of collecting interval. The semen samples were transported to the laboratory immediately after collection. Sperm samples were evaluated for sperm concentration and sperm motility. The semen samples with more than 100 million sperm per milliliter, a volume of more than 100 ml, and sperm motility of more than 70% were selected for the experiment.

### Semen Processing

The semen was diluted with Beltsville thawing solution (BTS) (modified BTS^®^, Kubus Co. Ltd., Madrid, Spain) extender supplemented with different concentrations of butaphosphan and cyanocobalamin combination (Octafos^®^, Octa Memorial Co., Ltd., Bangkok, Thailand) (1 ml included 100 mg butaphosphan and 0.05 mg cyanocobalamin) [0 (control), 0.1, 0.2, 0.3, 0.4, and 0.5%]. The diluted semen samples (3,000 × 10^4^ sperm/ml) were dispersed into 100 ml plastic tubes and equilibrated for 4 h at 16°C (Magapor^®^, Magapor S.L., Zaragoza, Spain). The sperm motility, sperm kinetic parameters, sperm viability, mitochondrial activity, acrosome integrity, and plasma membrane integrity were evaluated in the diluted semen after 4 (day 0), 72 (day 3), 120 (day 5), and 168 (day 7) h of storage.

### Sperm Evaluation

#### Computer-Assisted Sperm Analysis

The semen samples were evaluated for sperm motility and sperm kinetic parameters using the CASA system (SCA^®^, Proiser S.L., Valencia, Spain). Sperm kinetic parameters consisted of the curvilinear velocity (VCL), straight line velocity (VSL), average path velocity (VAP), linearity (LIN), straightness (STR), amplitude of lateral head (ALH), wobble (WOB), and beat cross frequency (BCF).

#### Sperm Morphology

##### Sperm viability

Sperm vitality was evaluated using SYBR-14/ethidium homodimer-1 (EthD-1) (Fertilight^®^, Sperm Viability Kit, Molecular Probes Europe, Leiden, Netherlands). Briefly, 10 μl of aliquot of the sperm sample was thoroughly mixed with 1 μl of 14-μM EthD-1 (Molecular Probes Inc., OR, USA) in 1 ml PBS and 2.7 μl of 0.38-μM SYBR-14 (Dead/Alive Kit; Molecular Probes Inc.) in 1 ml dimethyl sulfoxide (DMSO) at 37°C for 15 min. The sperm was placed on a glass slide and covered with a coverslip. Sperm membrane integrity was assessed using an epifluorescent microscope (CX-31; Olympus, Tokyo, Japan) at × 1,000 magnification. Two hundred sperm were evaluated in five different areas and classified into two categories: live and dead sperm which were stained only green from SYBR-14 (live) and stained both green and red or stained only red from EthD-1 (dead). The percentages of intact sperm membrane were calculated.

##### Mitochondrial activity

Sperm mitochondrial membrane was determined by using fluorochrome 5,5′,6,6′-tetrachloro-1,1′,3,3′-tetraethylbenzimidazoly-carbocyanine iodide (Molecular Probes, Molecular Probes Inc., Eugene, OR). JC-1 is considered to make it possible to distinguish the mitochondrial membrane potential status (high and low). Briefly, 12.5 μl of aliquot of the sperm sample was mixed with 25 μM final concentration of JC-1 in DMSO and then incubated in the dark at 37°C for 30 min. Counting of the sperm was conducted on individual spermatozoa until 200 sperm had been counted using an epifluorescent microscope (CX-31; Olympus, Tokyo, Japan) at × 1,000 magnification.

##### Acrosome integrity

Acrosome integrity was evaluated using EthD-1 (Fertilight^®^, Sperm Viability Kit, Molecular Probes Europe, Leiden, Netherlands). Briefly, 10 μl of aliquot of the sperm sample was thoroughly mixed with 10 μl of 14-μM EthD-1 (Molecular Probes Inc., OR, USA) at 37°C for 15 min. Five microliters of the mixture was placed on a glass slide and dropped into 95% ethyl alcohol for 30 s and then added to 15 μl FITC-PNA solution [FITC-PNA in PBS (1:10, v/v)] at 4°C for 30 min and removed by PBS. Acrosome integrity was assessed using an epifluorescent microscope (CX-31; Olympus, Tokyo, Japan) at × 1,000 magnification. Two hundred sperm were evaluated in five different areas.

##### Plasma membrane integrity

Sperm membrane integrity was determined using the short hypo-osmotic swelling test (sHOST). Briefly, 10 μl of aliquot of the sperm sample was thoroughly mixed with 200 μl citrate buffer (75 mOsm), incubated in the dark at 37°C for 30 min, and then, added to 175 μl Hos solution with 5% formaldehyde (75 mOsm). The sperm sample was placed on a glass slide and covered with a coverslip. Counting of the sperm was conducted on individual spermatozoa until 200 sperm had been counted under a light microscope (×400).

### Statistical Analysis

Statistical analyses were carried out by using SAS (SAS Institute, Cary, NC, USA). Sperm parameters including sperm motility, sperm kinetic parameters, sperm viability, acrosome integrity, mitochondrial activity, and functional membrane integrity were analyzed by using multiple analysis of variance (ANOVA). The analyses were carried out by using the general linear mixed model (MIXED) procedure of SAS. The statistical models for each data set included group (control, 0.1, 0.2, 0.3, 0.4, and 0.5% of butaphosphan and cyanocobalamin), day after storage (days 0, 3, 5, and 7), and interaction between group and day after collection. The boar was included as a random effect. Least square means were obtained from each class of the factor and were compared by using the least significant test (LSD). For all analyses, *P* < 0.05 was regarded to be statistically significant.

## Results

The levels of significance for sperm characteristics, day after collection, and interactions included in the statistical model are presented in Table 1. Sperm motility, all sperm kinetic parameters except ALH, sperm viability, and plasma membrane integrity were affected by butaphosphan and cyanocobalamin supplementation over the entire experimental period (Table 1). Sperm motility, all sperm kinetic parameters except ALH, sperm viability, and plasma membrane integrity in 0.3% of butaphosphan and cyanocobalamin supplementation were greater than those in the control group (Table 2). All sperm characteristics decreased during the day after collection (*P* < 0.001).

**Table 1:** Level of significance for sperm characteristic, day after collection, and interactions included in the statistical model using the MIXED procedure of SAS.

**Sperm characteristic**	**Group**	**Day**	**Group × Day**
Total motility, %	<0.001	<0.001	0.877
Progressive motility, %	<0.001	<0.001	0.862
VCL, μm/s	0.002	<0.001	0.927
VSL, μm/s	<0.001	<0.001	0.428
VAP, μm/s	<0.001	<0.001	0.892
LIN, %	<0.001	<0.001	0.114
STR, %	0.009	<0.001	0.264
WOB, %	<0.001	<0.001	0.258
ALH, μm	0.327	0.049	0.760
BCF, beats/s	<0.001	<0.001	0.242
Viability, %	0.018	<0.001	0.987
Acrosome, %	0.118	<0.001	0.717
Membrane, %	<0.001	<0.001	0.899
Mitochondria, %	0.058	<0.001	0.999

*VCL, curvilinear velocity; VSL, straight line velocity; VAP, average path velocity; LIN, linearity; STR, straightness; ALH, amplitude of lateral head; WOB, wobble; BCF, beat cross frequency*.

**Table 2:** Effect of 100 mg of butaphosphan and 0.05 mg of cyanocobalamin in different concentrations [0 (control), 0.1, 0.2, 0.3, 0.4, and 0.5%] and day after storage on semen characteristics from 35 ejaculates analyzed using the MIXED procedure of SAS.

**Parameters**	**Group**						SEM^*^	**Day**				**SEM**
	**Control**	**0.1**	**0.2**	**0.3**	**0.4**	**0.5**		**0**	**3**	**5**	**7**	
Total motility, %	60.7^c^	66.2^ab^	64.7^b^	**67.6^a^**	64.1^ab^	61.6^c^	2.4	73.7^a^	63.1^b^	61.7^b^	58.0^c^	2.4
PR, %	48.7^d^	54.0^ac^	52.5^bc^	**56.5^a^**	51.6^ce^	49.0^de^	2.5	62.2^a^	51.2^b^	49.3^b^	45.4^c^	2.5
VCL, μm/s	76.7^d^	80.1^abc^	79.2^bc^	**82.4**^**a**^	78.1^cd^	77.7^cd^	3.4	83.2^a^	78.9^b^	77.9^bc^	76.2^c^	3.4
VSL, μm/s	16.4^c^	17.6^ab^	17.2^bc^	**18.4^a^**	17.0^bc^	16.6^bc^	0.9	19.5^a^	17.4^b^	16.4^c^	15.4^d^	0.8
VAP, μm/s	35.6^c^	36.8^b^	36.1^b^	**38.3^a^**	36.0^bc^	35.4^bc^	1.8	37.7^a^	36.7^b^	35.8^b^	34.6^c^	1.8
LIN, %	20.6^d^	21.4^bc^	21.2^bcd^	**22.2^a^**	21.5^ac^	20.5^d^	0.6	23.0^a^	21.8^b^	20.6^c^	19.6^d^	0.6
STR, %	43.4^b^	44.2 ^ab^	44.1^ab^	**45.1^a^**	44.1^ab^	43.1^b^	1.0	48.2^a^	44.3^b^	42.3^c^	41.1^d^	0.9
WOB, %	43.6^c^	44.7^b^	44.3^bc^	**45.5^a^**	45.0^ab^	43.6^c^	0.6	44.0^a^	45.6^b^	44.7^b^	43.9^a^	0.6
ALH, μm	1.8	1.9	1.9	1.9	2.0	1.8	0.1	2.0^a^	1.9^ab^	1.9^b^	1.8^b^	0.1
BCF, beats/s	7.6^b^	8.1^ac^	8.0^b^	**8.4^a^**	7.8^bc^	7.7^b^	0.3	9.1^a^	8.0^b^	7.6^c^	7.0^d^	0.3
Viability, %	81.3^bc^	82.6^ac^	81.0^bc^	**83.4^a^**	81.8^bc^	82.0^ac^	1.0	85.2^a^	83.5^b^	81.1^c^	78.2^d^	1.0
Acrosome, %	84.4^ad^	**85.8^c^**	84.2^b^	84.8^abc^	84.6^bd^	84.9^bc^	0.9	86.0^a^	85.1^b^	84.3^bc^	83.8^c^	0.9
Membrane, %	42.4^d^	46.2^ac^	45.3^bc^	**46.9^a^**	44.2^b^	42.6^d^	1.9	52.0^a^	46.5^b^	42.0^c^	38.0^d^	1.8
Mitochondria, %	74.0^ac^	74.9^ab^	72.8^bc^	**76.2^a^**	72.2^bc^	74.6^ac^	1.9	77.8^a^	75.2^b^	73.3^b^	70.2^c^	1.8

a, b, c, d, e *Different superscript letters within rows indicate significant differences (P < 0.05)*.

* *Greatest standard error of the mean (SEM). The bold values provide the maximum values in each parameter*.

### Effects of Different Concentrations of Butaphosphan and Cyanocobalamin and Day of Storage on Sperm Motility

**Figure 1:**
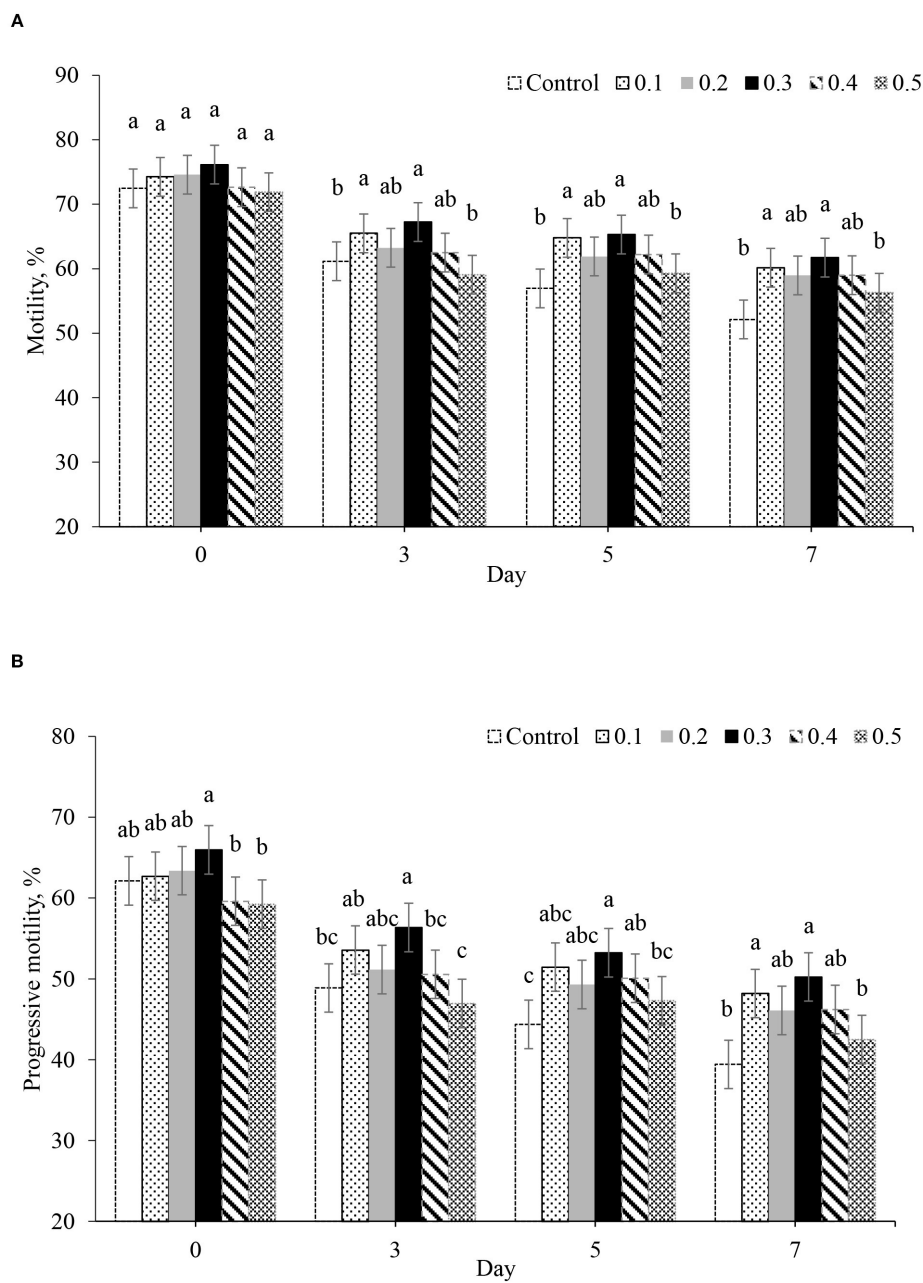
Effect of 100 mg of butaphosphan and 0.05 mg of cyanocobalamin in different concentrations [0 (control), 0.1, 0.2, 0.3, 0.4, and 0.5%] on total motility **(A)** and progressive motility **(B)** by day after storage (*n* = 35 ejaculations). ^a,b,c^Significant differences among groups in each day after storage (*P* < 0.05).

The total motility and progressive motility in 0.3% of butaphosphan and cyanocobalamin supplementation were highest in all of the day of collection. On day 0 after storage, no effect of butaphosphan and cyanocobalamin supplementation on total motility was found (Figure 1A). Progressive motility of boar sperm in 0.3% of butaphosphan and cyanocobalamin supplementation (66.0%) was greater than that in the 0.4% (59.6%, *P* = 0.024) and 0.5% supplementation (59.3%, *P* = 0.017) (Figure 1B). On days 3, 5, and 7 after storage, total motility and progressive motility in 0.3% of butaphosphan and cyanocobalamin supplementation were greater than those in the control (*P* < 0.05).

### Effects of Different Concentrations of Butaphosphan and Cyanocobalamin and Day of Storage on Sperm Kinetic Parameters

All sperm kinetic parameters were assessed by the CASA system on each day after collection (Tables 3, 4). The values of all sperm kinetic parameters decreased during storage. On day 0 after storage, the values for VCL, VSL, and VAP in 0.3% of butaphosphan and cyanocobalamin supplementation were greater than those in the 0.4 and 0.5% supplementation (*P* < 0.05) (Table 3). On day 3 after storage, the values for VCL, VSL, VAP, LIN, WOB, and BCF in 0.3% of butaphosphan and cyanocobalamin supplementation were greater than those in the control group (*P* < 0.05) (Table 3). On day 5 after storage, the values for VAP and WOB in 0.3% of butaphosphan and cyanocobalamin supplementation were greater than those in the control group (*P* < 0.05) (Table 4). On day 7 after storage, the values for all parameters except for ALH in 0.3% of butaphosphan and cyanocobalamin supplementation were greater than those in the control group (*P* < 0.05) (Table 4).

**Table 3:** Effect of 100 mg of butaphosphan and 0.05 mg of cyanocobalamin in different concentrations [0 (control), 0.1, 0.2, 0.3, 0.4, and 0.5%] on semen characteristics at days 0 and 3 after storage from 35 ejaculates.

**Parameters**	**Day 0**							**Day 3**						
	**Control**	**0.1**	**0.2**	**0.3**	**0.4**	**0.5**	SEM^*^	**Control**	**0.1**	**0.2**	**0.3**	**0.4**	**0.5**	**SEM**
VCL, μm/s	83.2^ab^	83.0^ab^	83.8^ab^	**88.1^a^**	80.4^b^	80.7^b^	3.8	76.9^b^	80.2^ab^	79.2^ab^	**82.8^a^**	77.4^ab^	76.8^b^	3.9
VSL, μm/s	19.9^ab^	19.2^b^	20.0^ab^	**21.1^a^**	18.2^b^	18.9^b^	1.0	16.2^b^	17.9^ab^	17.4^ab^	**18.5^a^**	17.7^ab^	16.8^ab^	1.0
VAP, μm/s	37.5^ab^	37.5^ab^	37.9^ab^	**40.3^a^**	36.3^b^	36.6^b^	2.0	35.0^b^	37.4^ab^	36.6^ab^	**38.9^a^**	36.5^ab^	35.8^b^	2.0
LIN, %	23.3	22.7	23.3	**23.5**	22.2	22.8	0.8	20.7^c^	21.7^abc^	21.8^abc^	22.3^ab^	**23.0^a^**	21.0^b^	0.8
STR, %	48.6	47.7	**48.9**	48.8	47.1	48.1	1.2	43.2^b^	44.4^ab^	44.6^ab^	45.1^ab^	**45.6^a^**	43.1^b^	1.2
WOB, %	43.8	43.8	44.0	**44.5**	43.6	43.9	0.8	44.3^b^	45.5^ab^	45.0^ab^	46.0^a^	**46.3^a^**	44.9^ab^	0.8
ALH, μm	1.9^b^	1.9^b^	1.9^b^	**2.0^b^**	2.4^a^	1.9^b^	0.1	1.8	1.9	1.9	1.9	1.9	1.8	0.1
BCF, beats/s	9.3^abc^	8.9^bc^	9.3^ac^	**9.7^a^**	8.6^b^	8.8^bc^	0.4	7.6^b^	8.3^ab^	8.1^ab^	**8.4^a^**	8.0^ab^	7.7^ab^	0.4

a, b, c *Different superscript letters within rows indicate significant differences (P < 0.05)*.

* *Greatest standard error of the mean (SEM). The bold values provide the maximum values in each parameter*.

**Table 4:** Effect of 100 mg of butaphosphan and 0.05 mg of cyanocobalamin in different concentrations [0 (control), 0.1, 0.2, 0.3, 0.4, and 0.5%] on semen characteristics at days 5 and 7 after storage from 35 ejaculates.

**Parameters**	**Day 5**							**Day 7**						
	**Con**.	**0.1**	**0.2**	**0.3**	**0.4**	**0.5**	SEM^*^	**Con**.	**0.1**	**0.2**	**0.3**	**0.4**	**0.5**	**SEM**
VCL, μm/s	75.3	78.2	78.0	**79.3**	78.6	77.8	3.9	71.5^b^	79.0^a^	75.8^ab^	**79.3^a^**	76.2^ab^	75.4^ab^	3.9
VSL, μm/s	15.6	16.4	16.3	**17.1**	17.1	16.1	2.0	13.8^b^	16.8^ac^	15.0^bc^	**16.9^a^**	15.1^abc^	14.8^b^	2.0
VAP, μm/s	34.1^b^	36.1^ab^	35.5^ab^	**37.1^a^**	36.7^ab^	35.5^ab^	2.0	31.8^b^	36.1^ac^	34.3^ab^	**37.0^a^**	34.6^ab^	33.9^bc^	2.0
LIN, %	20.1^ab^	20.6^ab^	20.2^ab^	**21.6^a^**	21.5^a^	19.9^b^	0.8	18.4^b^	20.6^ac^	19.6^bc^	**21.4^a^**	19.4^bc^	18.2^bc^	0.8
STR, %	42.0	42.0	42.2	**43.2**	43.0	41.6	1.2	39.8^b^	42.5^ac^	40.9^bc^	**43.1^a^**	40.5^bc^	39.5^b^	1.2
WOB, %	44.0^bc^	45.1^ac^	44.2^bc^	**46.1^a^**	45.9^a^	42.6^b^	0.8	42.4^c^	44.4^ab^	44.2^ab^	**45.5^a^**	44.1^ab^	42.9^bc^	0.8
ALH, μm	1.8	1.9	1.9	1.9	1.9	1.9	0.1	1.7	1.9	1.8	1.9	1.9	1.8	0.1
BCF, beats/s	7.2	7.7	7.6	**7.8**	7.8	7.5	0.4	6.3^b^	7.6^ac^	6.8^b^	**7.7^a^**	6.9^bc^	6.7^bc^	0.4

a, b, c *Different superscript letters within rows indicate significant differences (P < 0.05)*.

* *Greatest standard error of the mean (SEM). The bold values provide the maximum values in each parameter*.

### Effects of Different Concentrations of Butaphosphan and Cyanocobalamin and Day of Storage on Sperm Morphology

**Figure 2:**
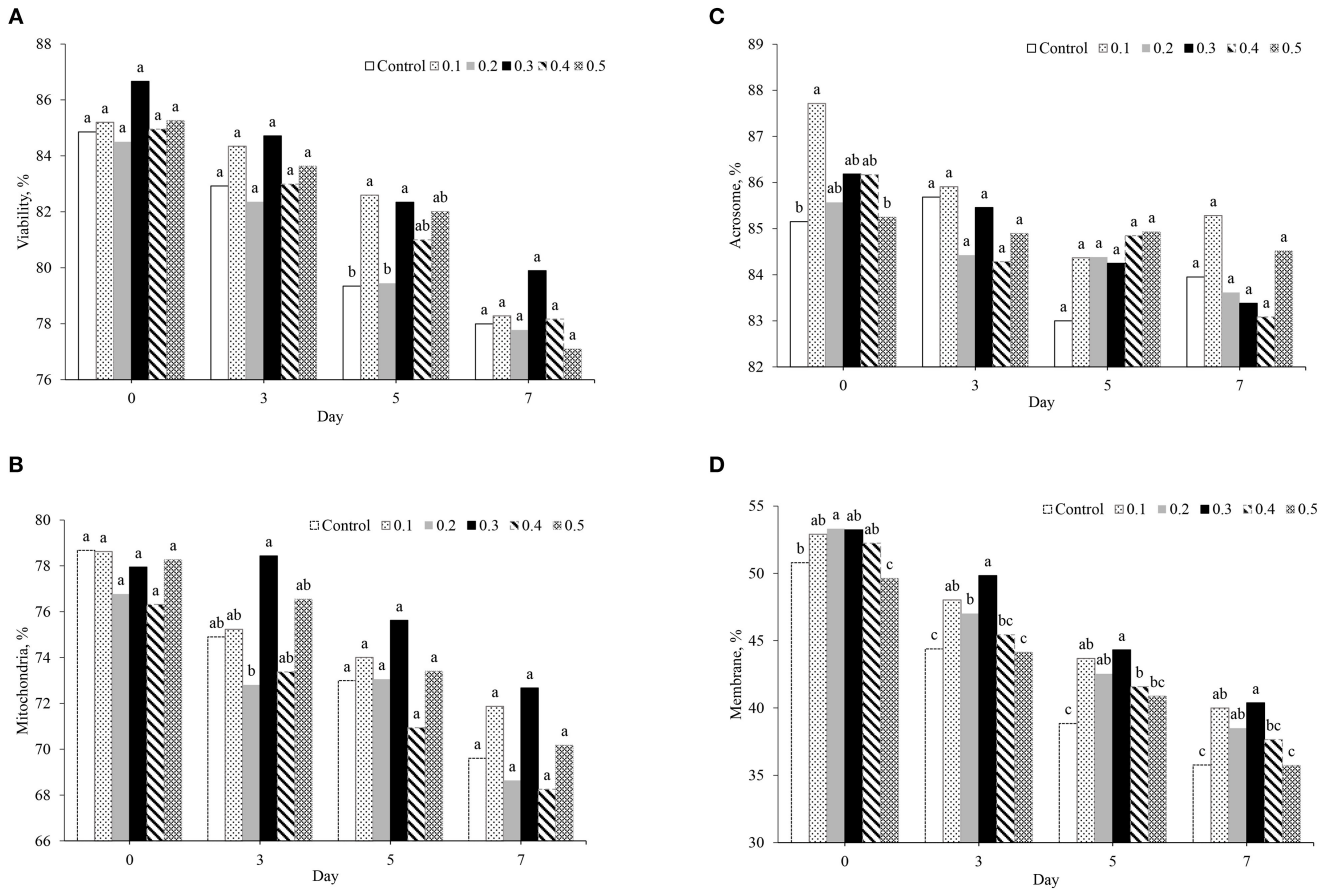
Effect of 100 mg of butaphosphan and 0.05 mg of cyanocobalamin in different concentrations [0 (control), 0.1, 0.2, 0.3, 0.4, and 0.5%] on sperm viability **(A)**, mitochondria activity **(B)**, acrosome integrity **(C)**, and plasma membrane integrity **(D)** by day after storage (*n* = 35 ejaculations). ^a,b,c^Significant differences among groups on each day after storage (*P* < 0.05).

The effect of butaphosphan and cyanocobalamin supplementation on sperm quality, assessed by fluorescence staining in different extenders, is presented in Figure 2. On day 0 after storage, no effect of butaphosphan and cyanocobalamin supplementation on sperm viability and mitochondria activity was found (Figures 2A,B). Acrosome activity was higher in 0.1% of butaphosphan and cyanocobalamin supplementation (87.7%) and was greater than that in the control (85.2%, *P* = 0.028) and in the 0.5% supplementation (85.2%, *P* = 0.034) (Figure 2C). Moreover, sperm membrane permeability in 0.3% of butaphosphan and cyanocobalamin supplementation (53.2%) was greater than that in the 0.5% (49.6%, *P* = 0.005) and had a tendency to be higher than that in the 0.4% supplementation (50.8%, *P* = 0.060) (Figure 2C). On day 3 after storage, no effects of 0.3% of butaphosphan and cyanocobalamin supplementation on sperm viability, acrosome integrity, and mitochondria activity were found. Sperm plasma membrane integrity in 0.3% of butaphosphan and cyanocobalamin supplementation was greater than that in the 0.5% and the control group on days 3, 5, and 7 after storage (*P* < 0.001) (Figure 2D). On day 5 after storage, sperm viability in 0.1 and 0.3% of butaphosphan and cyanocobalamin supplementation was significantly greater than that in the 0.2% and the control group. No effects of butaphosphan and cyanocobalamin supplementation on sperm viability, acrosome integrity, and mitochondria activity were found on day 7 after storage.

## Discussion

The present study is the first report to provide information on the effects of an injectable product including butaphosphan and cyanocobalamin supplementation in chilled boar semen extender on sperm quality and life span. Our results indicated that 0.3% of butaphosphan and cyanocobalamin supplementation in the chilled boar semen extender increased sperm motility, sperm kinetic parameters, and sperm morphology. Therefore, butaphosphan and cyanocobalamin supplementation in semen extender improved chilled boar sperm quality and life span and may be applied in commercial swine herds. This finding will improve our knowledge in improving sperm quality in chilled boar semen and could be used to modify injectable products to be used in chilled boar semen to improved swine fertility.

### Effect of Butaphosphan and Cyanocobalamin Supplementation on Sperm Quality

Butaphosphan or phosphorus plays an important role in increasing sperm motility because phosphorus has a crucial role in sperm energy metabolism including ATP and AMP, the production of creatinine and nucleotides, gluconeogenesis, and glycogenesis (4, 5). Moreover, phosphorus stimulates protein function in phosphorylation (16). López Rodríguez et al. (17) found that phosphate concentration in seminal plasma positively correlated with sperm concentration and sperm motility in boars. Furthermore, cyanocobalamin involves energy and glucose metabolism. Cyanocobalamin is a cofactor of methylmalonyl-CoA mutase which is used to convert propionate to succinyl-CoA in the Krebs cycle (6) and is used in gluconeogenesis (7). The results of the present study demonstrated that all concentrations of butaphosphan and cyanocobalamin improved both sperm total motility and progressive motility and the values of VCL, VSL, VAP, LIN, STR, WOB, and BCF. In agreement with Beltrama et al. (18), an intramuscular injection of butaphosphan and cyanocobalamin combination increased sperm motility in mice. Sperm viability and membrane integrity were increased by butaphosphan and cyanocobalamin supplementation in chilled boar semen. From our results, 0.3% of butaphosphan and cyanocobalamin supplementation increased 2.1% of sperm viability and 4.5% of sperm plasma membrane integrity in the chilled boar semen extender and increased semen quality when compared with the control group. Similarly, many previous studies have reported the effect of cyanocobalamin supplementation during the thawing of frozen semen on semen quality and fertilization in many species (8, 9, 19, 20). In boars, supplementation of 0.5 and 1.0 μg cyanocobalamin increased progressive sperm motility and plasma membrane viability (20). In rams, Hamedani et al. (9) supplemented 2.0 mg/ml of cyanocobalamin in the extender preserved at 5°C, and it improved sperm motility, viability, the number of normal sperm, and plasma membrane viability in pre- and post-freezing conditions. Moreover, in Hu et al. (19), the supplementation of 2.5 mg/ml of cyanocobalamin during post-thawing increased the sperm quality. However, Beltrama et al. (18) found that an intramuscular injection of butaphosphan and cyanocobalamin supplementation in mice improved mitochondria activity and acrosome integrity. This contrasts with our results, in which the supplementation of butaphosphan and cyanocobalamin did not result in improvement. The intramuscular injection of the combination of butaphosphan and cyanocobalamin administered exerts a potential role in spermatogenesis and structure, while supplementation in the extender increases sperm activity.

Increasing sperm quality by butaphosphan and cyanocobalamin supplementation improved fertility in swine herds. The values of VSL, VAP, LIN, and STR were positively correlated with litter size in pig (21) and with fertility in humans (22, 23). Additionally, cyanocobalamin impairs reactive oxygen species (ROS) and positively relates with sperm quality, concentration, and fertility rates in humans (24, 25). In accordance with Barranco et al. (26), who measured the total antioxidant capacity in seminal plasma in boars, they found that individual total antioxidant capacity in boars was greatly varied. Moreover, the total antioxidant capacity in seminal plasma in boars positively correlated with sperm concentration, conception rate, and fertility. Therefore, it can be concluded that the beneficial effects of butaphosphan and cyanocobalamin supplementation in chilled boar semen were increased energy and increased functionality of the plasma membrane leading to improved semen quality in boars.

### Effect of Butaphosphan and Cyanocobalamin Supplementation on Semen Life Span

Semen preservation by an extender has been widely used for enhancing semen life span. The extender provides the preserved sperm cells and components, source of energy, proper pH and osmotic pressure depended on the ingredients of the sperm preservation. The short time preservation [i.e., BTS, Illinois variable temperature (IVT), and Kiev] can preserve sperm for about 1–3 days. The BTS is generally used in the swine production industry due to its inexpensiveness, ease of use, and appropriated preservation time. Therefore, BTS was used to preserve semen in the present study. Free radicals gradually increased from oxidative stress conditions such as stresses during cooling and storage time (27), which could damage membrane structure and mitochondria function (28). Sperm membranes are rich in polyunsaturated fatty acids (PUFAs), which are highly sensitive to lipid peroxidation. Sperm membranes were destroyed by lipid peroxidation, leading to leaking of sperm intracellular organisms and inhibiting the respiratory systems of the sperm cell (29). Moreover, free radicals in sperm cells declined ATP utilization at the contractile apparatus of the flagellum (30). Sperm cells rapidly decreased motility and death from lipid peroxidation (31). Butaphosphan may provide energy reserves and cyanocobalamin protects sperm cells from ROS during storage at low temperatures (24), thereby increasing the sperm motility and life span. The present study demonstrated that sperm in 0.3% of butaphosphan and cyanocobalamin supplementation has a higher percentage of viability than that in the control group at day 5 of storage. Similarly, a previous study found that supplementation with vitamin B_12_ in bull cow semen increases semen quality and increases semen lifetime (32). In agreement with our results, sperm motility, sperm viability, and plasma membrane integrity increased after day 3 of storage. In general, above 60% of sperm total motility after dilution was used in AI in swine herds. At day 5 of storage, semen with 0.1–0.4% butaphosphan and cyanocobalamin supplementation in chilled boar semen had total motility above 60%, whereas the control group and 0.5% supplementation had total motility below 60% in the present study. Moreover, semen with 0.1 and 0.3% butaphosphan and cyanocobalamin supplementation in chilled boar semen still had total motility above 60% at day 7 of storage. Additionally, concerning sperm morphology, semen with 0.1 and 0.3% butaphosphan and cyanocobalamin supplementation has significantly higher viability than in the control group at day 5 after storage and has higher plasma membrane integrity at day 7 after storage. Therefore, it can be concluded that the supplementation with 0.3% butaphosphan and cyanocobalamin in the extender could significantly enhance semen quality and prolong the life span period of the sperm.

High concentrations of butaphosphan and cyanocobalamin supplementation have an adverse effect on semen. Hu et al. (8) reported that 3.75 mg/ml of cyanocobalamin supplementation reduced cow semen quality. The present study found that semen with 0.4 and 0.5% of butaphosphan and cyanocobalamin supplementation (equivalent to 0.020 and 0.025 mg/ml, respectively) did not improve semen quality and life span when compared with the control group. The mechanism of butaphosphan and cyanocobalamin supplementation having an adverse effect on semen quality is still not clear. Inappropriate antioxidative supplementation may cause increased cell death because antioxidants cannot distinguish between advantageous and disadvantageous radicals. High antioxidant supplementation acts as prooxidants by increasing oxidative stress and disturbs the ROS formation and neutralization balance (33). It can be concluded that supplementation with butaphosphan and cyanocobalamin in an extender should not be used in excess of 0.02 mg/ml.

## Conclusions

In conclusion, the beneficial effects of 0.3% butaphosphan and cyanocobalamin supplementation on chilled boar semen were increased energy and increased functionality of the plasma membrane leading to improved semen quality, sperm activity, morphology, and life span of chilled boar sperm.

## Data Availability Statement

All datasets generated for this study are included in the article/supplementary material.

## Ethics Statement

The present study was approved by the Chulalongkorn University Animal Care and Use Committee (animal use protocol number 1831110). The protocols followed the guidelines documented in the ethical principles and guidelines for the use of animals for scientific purposes published by the National Research Council of Thailand. Written informed consent was obtained from the owners for the participation of their animals in this study.

## Author Contributions

All authors listed have made a substantial, direct and intellectual contribution to the work, and approved it for publication.

## Conflict of Interest

The authors declare that the research was conducted in the absence of any commercial or financial relationships that could be construed as a potential conflict of interest.
